# Place of robotic surgery in completion lobectomy after anatomical segmentectomy

**DOI:** 10.1093/icvts/ivad137

**Published:** 2023-08-12

**Authors:** Juliette Piccoli, Joseph Seitlinger, Arthur Streit, Christophe Wollbrett, Joelle Siat, Stéphane Renaud

**Affiliations:** Department of Cardiac Surgery, Nancy Regional University Hospital, Institut Lorrain du Cœur et des Vaisseaux. Rue du Morvan, Vandœuvre-lès-Nancy, France; Department of Thoracic Surgery, Nancy Regional University Hospital, Institut Lorrain du Cœur et des Vaisseaux. Rue du Morvan, Vandœuvre-lès-Nancy, France; Department of Thoracic Surgery, Nancy Regional University Hospital, Institut Lorrain du Cœur et des Vaisseaux. Rue du Morvan, Vandœuvre-lès-Nancy, France; Department of Thoracic Surgery, Nancy Regional University Hospital, Institut Lorrain du Cœur et des Vaisseaux. Rue du Morvan, Vandœuvre-lès-Nancy, France; Department of Thoracic Surgery, Nancy Regional University Hospital, Institut Lorrain du Cœur et des Vaisseaux. Rue du Morvan, Vandœuvre-lès-Nancy, France; Department of Thoracic Surgery, Nancy Regional University Hospital, Institut Lorrain du Cœur et des Vaisseaux. Rue du Morvan, Vandœuvre-lès-Nancy, France

**Keywords:** Completion lobectomy, Segmentectomy, Robot-assisted thoracic surgery, Thoracic surgery

## Abstract

**OBJECTIVES:**

Although segmentectomy is steadily increasing in early-stage non-small-cell lung cancer, recurrence in the ipsilateral lobe is also increasing. Completion lobectomy (CL) is a challenging procedure that has already been described in a few studies using video-assisted thoracic surgery or thoracotomy. In this study, we aimed to show the feasibility and safety of robot-assisted thoracic surgery in cases of CL.

**METHODS:**

Among 2073 major resections performed between January 2018 and september 2022 in the Department of Thoracic Surgery at Nancy University Regional Hospital, we retrospectively included patients who underwent CL by robot-assisted thoracic surgery after previous segmentectomy for non-small-cell lung cancer. Data and perioperative results were described and analysed.

**RESULTS:**

Seventeen patients underwent CL with a median recurrence time after previous segmentectomy of 18 months [interquartile range (IQR): 12]. Four patients (23.5%) had a pulmonary artery injury that was controlled, and no conversion to open thoracotomy was needed. The operative time was 150 min (IQR: 20), and blood loss was 300 ml (IQR: 150). The median postoperative chest tube duration was 2 days (IQR: 1), and the length of hospital stay was 3 days (IQR: 3), with no postoperative deaths.

**CONCLUSIONS:**

Completion lobectomy is a challenging procedure due to severe adhesions surrounding vessels, which potentially could cause higher rate of PA bleeding than conventional surgeries. With experienced team and surgeons, CL with robotic surgery may be reported as a safe and feasible procedure.

**Figure:**
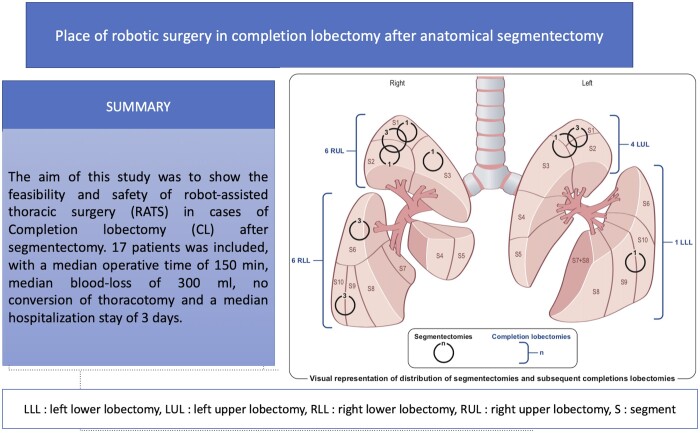

## INTRODUCTION

To date, surgery remains the cornerstone of treatment in early-stage non-small-cell lung cancer (NSCLC). In the last decade, there has been a shift in the surgical management of tumours <2 cm, with segmentectomy overcoming lobectomy. The place of pulmonary segmentectomy will probably steadily increase in the coming years, thanks to the results of the JCOG0802 and CALGB 140503 studies [1, 2]. Indeed, the authors showed the benefits of segmentectomy in overall survival in patients with clinical stage IA NSCLC compared to lobectomy and suggested that segmentectomy should become the standard treatment. However, while offering better preservation of lung function compared to lobectomy, there was more recurrence in the ipsilateral remaining lobe after segmentectomy [3, 4]. In these cases, completion lobectomy (CL) remains the best option in operable patients. However, CL is usually challenging due to previous surgeries and related adhesions surrounding the vessels. Previous authors, based on small cohort studies, have already focused on this, highlighting the technical difficulties of video-assisted thoracic surgery (VATS) CL. Nevertheless, these studies showed the feasibility of the technique in experienced centres in minimally invasive surgery [5–10].

In recent years, robot-assisted surgery has gained a place in oncological resections, including thoracic malignancies. In thoracic surgery, many studies have shown its equivalence to VATS [11, 12]. Indeed, advantages of minimally invasive surgery, such as reduction of blood loss, postoperative complications, and shorter length of hospitalization, have been demonstrated [13, 14]. Moreover, robot-assisted thoracic surgery (RATS) offers surgeons great comfort due to its 3D high-definition view, tremor filtration, better dexterity, and ergonomic maneuverability of instruments with 7 degrees of freedom [15, 16]. Meanwhile, RATS is commonly used in experienced centres, and no study has focused thus far on robotic surgery in CL.

The aim of this study was to assess the place of RATS in the CL after lung anatomical segmentectomy.

## MATERIALS AND METHODS

### Ethics statement

This study was approved by the Ethics Committee of the French Society of Thoracic and Cardiovascular Surgeons (Approval Number: IRB00012919). Written consent to exploit data was obtained for each patient.

### Covariates and data collection

This is a single-centre retrospective cohort study performed in the Department of Thoracic Surgery at Nancy University Regional Hospital (Nancy, France) between January 2018 and September 2022.

In this time period, 2073 patients undergo a major lung resection for lung cancer in the Department of Thoracic Surgery at Nancy Regional University Hospital. Among them, 491 benefit from an anatomical segmentectomy with VATS or RATS.

**Figure 1:**
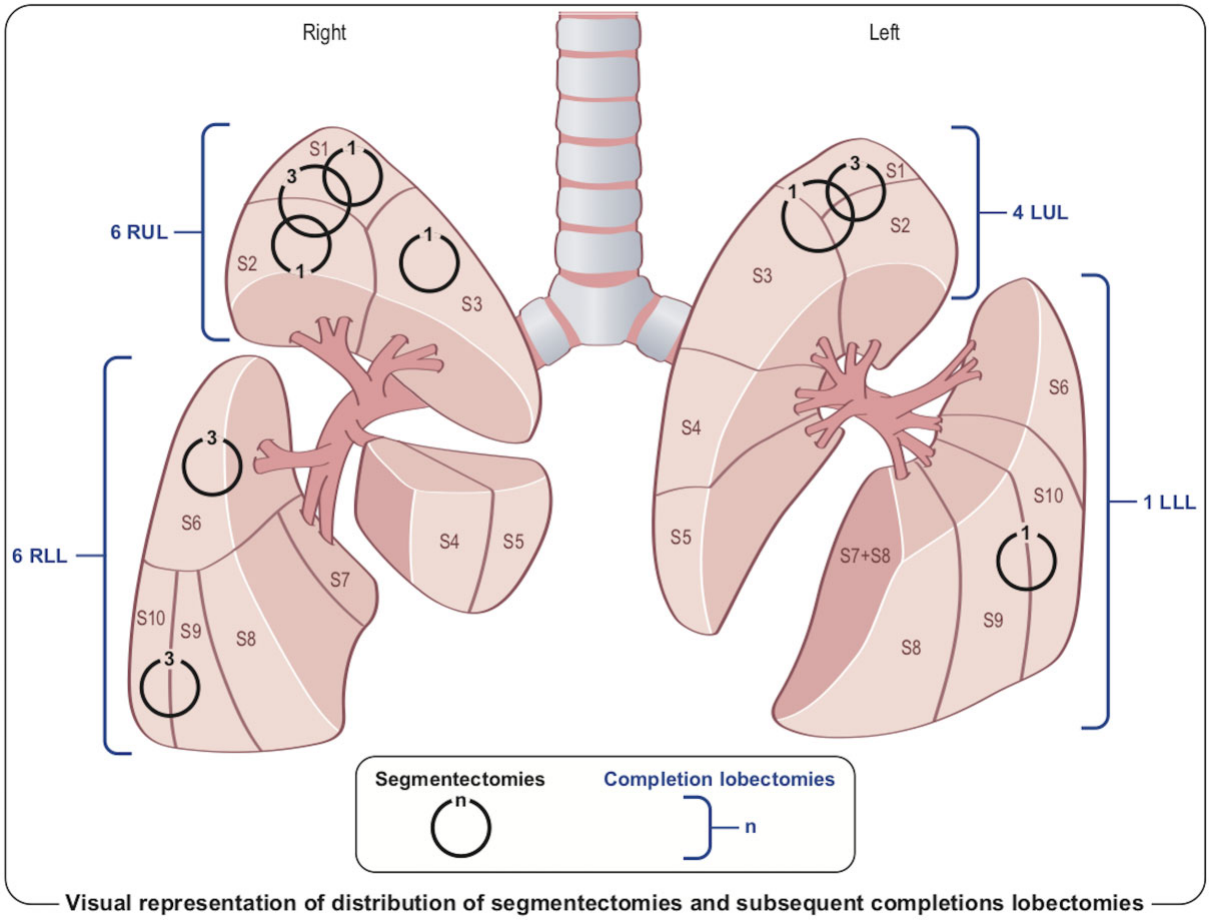
Distribution of segmentectomies and subsequent completions lobectomies.

We retrospectively reviewed data from patients over 18 years of age who benefitted from an anatomical segmentectomy, either by VATS or RATS, for stage IA NSCLC (8th edition of TNM staging system [17]). Segmentectomy was performed in cN0 patients on preoperative staging, with peripheral tumour <2 cm, distant from the visceral pleura. In this time period, all CL in our department of thoracic surgery were performed by RATS and were included in our study (Fig. 1). Recurrence was defined by CT appearance and morphological or metabolic modifications of a nodule in the remaining lobe.

For each patient, smoking status, sex, World Health Organization performance status and the Charlson Comorbidity Index, which incorporates 19 chronic diseases weighted according to their association with mortality, were calculated. We grouped patients into the following established categories according to their total Charlson Comorbidity Index score: 0 (no comorbidity); 1–2 (average), 3–4 (moderate), >5 (severe), time to recurrence (defined as the time elapsed between the first segmentectomy and radiological diagnosis of recurrence), distance between the recurrent nodule and the staple line, operative time of CL (defined as surgeon console time), pathological results, perioperative complications and blood loss, postoperative air leak duration, chest tube duration, hospital length of stay and postoperative mortality (any death, regardless of cause, within 30 days after surgery).

### Surgical approach

All patients were operated on by the same surgeon experienced in both VATS and RATS.

In the case of VATS, a biportal anterior approach was performed. A 2- to 3-cm incision was performed in the 4th intercostal space in the mid-axillary line. A camera port was inserted in the 7th or 8th intercostal space under vision control between the mid and posterior axillary lines.

In the case of RATS, Da Vinci Surgical system [Si from 2018 to 2019, and then Xi (Intuitive Surgical, Sunnyvale, CA, USA)] was used. A four-arm approach with an assistant port was preferred, with CO_2_ pressure at 9–12 mmHg. Robotic ports were inserted in the 7th intercostal space. The assistant port was placed anteriorly, just above the diaphragm, under vision control according to the placement of robotic ports to limit conflicts with robotic arms.

The surgical technique was always the same regardless of the approach. Before the first segmentectomy, systematic surgical planification was performed using Fuji Synapse 3D (Minato-ku, Tokyo, Japan) to ensure correct margins (20 mm or at least the nodule size) and avoid anatomical mistakes. During the primary segmentectomy, systematic lymph node dissection (LND) was always performed, including at stations 7, 9, 10 and 11 on both sides, station 5 on the left side and station 4 on the right side. Veins, arteries and bronchi were divided separately. Before parenchymal stapling at the end of the procedure, intravenous indocyanine green was injected to define the anatomical segmental plane.

During CL, an RATS approach was always performed. Ports’ placement was performed as described above, regardless of the location of CL. In this case, adhesions were first completely freed in the whole pleural cavity. Exposition of the anterior and posterior sides of the hilum was then performed, ensuring the main pulmonary artery (PA) was completely freed and controllable. The remaining lymph nodes were harvested. Depending on surgical findings, particularly when adhesions in the fissure were dense, a fissure less-fissure last approach was sometimes performed. The vein, bronchus and artery were usually stapled separately. However, in 3 lower CLs, because of dense adhesions between the artery and the parenchyma, they were both stapled together.

In all surgeries (both segmentectomies and CL), Progel (BD Franklin Lakes, USA) pleural air leak sealant was systematically applied on the staple lines and area of dissection to decrease the risk of postoperative air leak.

## RESULTS

According to our inclusion criteria, we finally focused on 17 patients. Our population was mainly composed of males (82%), with a median age of 69 years old [interquartile range (IQR): 3]. All patients, except one, were current smokers, with a median tobacco consumption of 20 (IQR: 8) pack years. The main approach during the first segmentectomy was RATS (65%). Histopathological analysis revealed 82% adenocarcinoma (ADK) and 18% squamous cell carcinoma (SCC). All resections were R0 (no small margins resections were noted either), and no patient received adjuvant treatment. Patient characteristics are disclosed in Table 1.

**Table 1:** Patient characteristics at first segmentectomy

No	Age/sex	Pack-year	WHO performance status	Charlson comorbidity Index	Side of first surgery left (L) or right (R)	First surgery approach: VATS/RATS	Segmentectomy	Postoperative histology: ADK/SCC	pTNM	Number of LN
1	69/F	25	1	1	L	VATS	S1-2-3	ADK	pT1bN0M0	18
2	58/M	20	1	1	R	RATS	S9-10	ADK	pT1bN0M0	22
3	72/M	30	2	1	R	RATS	S6	ADK	pT1bN0M0	16
4	69/M	25	1	1	L	VATS	S1-2	SCC	pT1bN0M0	24
5	71/M	22	2	1	R	RATS	S1-2	SCC	pT1aN0M0	15
6	70/M	15	2	2	R	VATS	S2	ADK	pT1bN0M0	21
7	78/M	30	2	2	R	VATS	S1-2	ADK	pT1aN0M0	17
8	69/M	55^a^	1	2	R	RATS	S9-10	ADK	pT1bN0M0	25
9	67/M	12	1	2	L	RATS	S9-10	ADK	pT1aN0M0	14
10	65/M	22	1	2	R	RATS	S1-2	ADK	pT1aN0M0	20
11	76/F	18	1	1	R	RATS	S3	ADK	pT1aN0M0	18
12	70/M	10	2	2	L	RATS	S1-2	ADK	pT1aN0M0	23
13	72/M	17	2	2	L	RATS	S1-2	ADK	pT1bN0M0	22
14	68/M	15	2	2	R	VATS	S6	ADK	pT1bN0M0	24
15	63/M	22	1	2	R	RATS	S6	SCC	pT1bN0M0	19
16	70/M	20	1	2	R	RATS	S9-10	ADK	pT1bN0M0	21
17	69/M	20	1	1	R	VATS	S1	ADK	pT1bN0M0	23

a This patient is a former smoker.

ADK: adenocarcinoma; LN: lymph nodes; RATS: robot-assisted thoracic surgery; SCC: squamous cell carcinoma; VATS: video-assisted thoracic surgery; WHO: World Health Organization.

The median recurrence time in the ipsilateral remaining lobe after segmentectomy was 18 months (IQR: 12). The median distance between the recurrent nodule and staple line was 20 mm (IQR: 7). The median operative console time was 150 min (IQR: 20). Injury of the PA was observed in 4 patients (23.5%). However, none of them occurred on the main PA. Injury of the PA branch was controlled with simple compression in 2 cases. In the 2 other cases, PA bleeding came from a branch of the PA. In those last cases, bleeding control was obtained by hem-o-lok and section of the branch. No conversion to open thoracotomy was necessary. The median total blood loss was 300 ml (IQR: 150). Resection after CL was R0 in the whole cohort. The median air-leak duration was 1 day (IQR: 1). The median chest tube duration was 2 days (IQR: 1). The length of hospital stay was 3 days (IQR: 3). Data on completion lobectomies are disclosed in Table 2.

**Table 2:** Outcomes of completion lobectomy

No	Time to recurrence (months)	Distance to staple line (mm)	Site of CL	Postop histology	Type of cancer	pTNM	Number of LN	Operative time (min)	PA injury	Blood loss (ml)	Postoperative complications	Air-leak duration (days)	Drainage duration (days)	Length of hospitalization (days)
1	12	18	LUL	ADK	Sc	pT1bN0M0	12	180	1	500	AF	1	2	4
2	18	20	RLL^a^	ADK	Sc	pT1cN0M0	11	140	0	300	0	2	3	3
3	12	20	RLL	ADK	Rc	pT1aN0M0	9	160	0	200	0	2	3	3
4	9	18	LUL	ADK	Sc	pT2aN1M0	10	200	1	500	Persistent air leak	7	8	9
5	12	9	RUL	SCC	Rc	pT1aN0M0	7	160	0	400		2	3	3
6	18	18	RUL	ADK	Sc	pT1bN1M0	9	120	0	300		1	2	2
7	24	25	RUL	ADK	Sc	pT2aN0M0	7	140	0	250	Ipsilateral pneumonia	1	2	5
8	24	30	RLL^a^	ADK	Sc	pT1cN0M0	12	140	0	300	0	0	2	2
9	18	25	LLL^a^	ADK	Rc	pT1cN0M0	11	120	0	300	Ipsilateral pneumonia	3	4	5
10	24	30	RUL	ADK	Rc	pT1cN0M0	9	150	0	200	0	1	2	2
11	12	15	RUL	ADK	Sc	pT1cN0M0	10	150	0	350	0	2	3	3
12	24	20	LUL	ADK	Rc	pT1cN0M0	6	180	1	800	Ipsilateral pneumonia	1	2	5
13	12	20	LUL	ADK	Sc	pT1cN0M0	8	150	0	350		3	4	4
14	12	25	RLL	ADK	Sc	pT2aN0M0	10	200	1	1200	AF	1	2	4
15	24	20	RLL	ADK	Sc	pT1bN0M0	7	150	0	200		1	2	2
16	24	15	RLL	ADK	Sc	pT1bN0M0	9	120	0	200		0	1	2
17	12	20	RUL	ADK	Rc	pT1bN0M0	10	150	0	250	Persistent air leak	9	10	11

a Stapling of the artery and the parenchyma all together.

ADK: adenocarcinoma, AF: atrial fibrillation, CL: completion lobectomy; LLL: left lower lobe, LN: lymph nodes, LUL: left upper lobe, PA: pulmonary artery, Rc: recurrence, RLL: right lower lobe, RUL: right upper lobe, Sc: secondary cancer, SCC: squamous cell carcinoma.

Overall postoperative morbidity was 41.1% (*n* = 7), represented by 2 atrial fibrillations (28.6%), 2 persistent (28.6%) air leaks and 3 postoperative pneumonia (42.8%). Three patients (patients 1, 4 and 17) had apical residual space on follow-up chest X-ray, resolving spontaneously in <3 weeks. No readmission was necessary, there were no postoperative deaths or delayed broncho-pleural fistula.

## DISCUSSION

Studies on CL after previous segmentectomy have already been published by several teams. However, they were all based on small populations, ranging from 4 to 11 patients. Furthermore, these studies only focused on VATS and open thoracotomy [5–10], showing the safety and feasibility of this technique in these 2 approaches. Nevertheless, the large majority of these studies have included patients with open thoracotomies, and data on minimally invasive approaches are poor. Finally, thus far, no data on robotic approaches have been published.

To our knowledge, we are here reporting the first study of CL using a RATS approach after segmentectomy in NSCLC, with thus far the largest published minimally invasive cohort.

As published by previous authors, although feasible in experienced hands, CL is a challenging technique. In particular, hilar adhesions following the first surgery, especially around the PA, can lead to massive bleeding during CL. Hence, Omasa *et al.* questioned the impact of the time period between the 2 surgeries on the occurrence of hilar adhesions. They divided patients into 2 groups according to the interval between segmentectomy and CL (group A 3–35 days, group B 56–1470 days) and observed more severe hilar adhesions in Group B with more PA injury [6]. They concluded that it was better if CL occurred within 5 weeks after the previous segmentectomy. However, the majority of patients in this cohort [6–11] underwent CL for complications in the remaining lobe or positive lymph nodes at the final pathological examination. In our study, the median recurrence time in the ipsilateral lobe after segmentectomy was 18 months, which reflects more what can be seen in real-life practice. LND, particularly in the mediastinum, is one of the causes of intense adhesions to the blood vessels, particularly the PA, increasing the risk of PA injury in cases of upper lobe CL on both sides. Takahashi *et al.* [9] chose as inclusion criteria patients with no radical LND of 4R during a previous segmentectomy of the right upper CL and LND of 5 and/or 4L during a previous segmentectomy of the left upper CL because of adhesions. In our cohort, all patients underwent a previous radical LND, including patients who underwent CL on the upper lobe. We emphasize that in these cases, dissection of the PA was more difficult, in particular because of dense adhesions between the bronchus and the PA and the parenchymal staple line and the PA. Indeed, 3 out of the 4 cases of PA injury were noted during left upper lobe CL. However, none of these cases required conversion to open thoracotomy to control the bleeding. Another way to ward off severe adhesions has been to staple the parenchyma with the PA, which is similar to other studies [5, 18]. On the other hand, some authors [5, 9] reported a systematic taping of the main PA, allowing its fast clamping in case of major bleeding. Indeed, because of the thin wall of the PA, it is exposed to a higher risk of rupture following dissection compared to the vein. In our experience, taping was not performed. However, we ensured first that the main PA was completely freed from adhesions to be able to clamp it. Nevertheless, whatever the used technique, it appears that ensuring a possible clamping of the main in CL is mandatory. Indeed, if major bleeding occurs, the time extension to the conversion to thoracotomy, delay and difficulty in securing the main PA due to adhesions particularly after upper lobe segmentectomy might increase the amount of bleeding.

A systematic literature review conducted by Liu *et al.* [10] compared median blood loss in VATS (229 ml) and thoracotomy (381 ml) approaches and showed no significant difference [5–9]. The blood loss in our cohort was fully comparable to these results.

Interestingly, the operative time in our cohort was shorter than those previously published (median console time: 150 min). Indeed, previous authors reported a median operative time of 272 min in VATS and 253 min in thoracotomy [10]. This could be linked not only to a finer and faster dissection in RATS but also to easier exposition compared to VATS.

However, this study has some limitations that must be taken into account. First, this is a single-centre study based on a small cohort, although this is the largest published study on minimally invasive surgery. However, our study is strengthened by the fact that no patients were excluded owing to the type of first segmentectomy, hence reflecting real-life practice. Furthermore, data can be biased by the fact that all patients were operated on by the same experienced surgeon in both VATS and RATS, limiting their generalizability to a wider population. Finally, because of its retrospective nature, some data, such as blood loss or operative time, could have been misestimated.

In conclusion, despite the difficulty of this technique, RATS in CL after segmentectomy seems to be a good alternative to open surgery and to VATS in experienced hands. Indeed, thanks to its several advantages [13], RATS seems to be related to good outcomes, with low perioperative and postoperative morbidity. However, our results must be interpreted with caution regarding the limitations listed above. Further studies on larger cohorts are needed to confirm these preliminary results.

**Conflict of interest:** none declared.

## Data Availability

All relevant data are within the article and its supporting information files.

## ABBREVIATIONS

ADK: Adenocarcinoma
CL: Completion lobectomy
IQR: Interquartile range
LND: Lymph node dissection
NSCLC: Non-small-cell lung cancer
PA: Pulmonary artery
RATS: Robot-assisted thoracic surgery
SCC: Squamous cell carcinoma
VATS: Video-assisted thoracic surgery
